# Modelling of Muscle Force Distributions During Barefoot and Shod Running

**DOI:** 10.1515/hukin-2015-0057

**Published:** 2015-10-14

**Authors:** Jonathan Sinclair, Stephen Atkins, Jim Richards, Hayley Vincent

**Affiliations:** 1 Division of Sport Exercise and Nutritional Sciences, School of Sport Tourism and Outdoors, University of Central Lancashire UK.; 2 Allied Health Research Unit, School of Sport Tourism and Outdoors UK, University of Central Lancashire, UK.

**Keywords:** Barefoot, running, muscle forces, kinematics

## Abstract

Research interest in barefoot running has expanded considerably in recent years, based around the notion that running without shoes is associated with a reduced incidence of chronic injuries. The aim of the current investigation was to examine the differences in the forces produced by different skeletal muscles during barefoot and shod running. Fifteen male participants ran at 4.0 m·s-1 (± 5%). Kinematics were measured using an eight camera motion analysis system alongside ground reaction force parameters. Differences in sagittal plane kinematics and muscle forces between footwear conditions were examined using repeated measures or Freidman’s ANOVA. The kinematic analysis showed that the shod condition was associated with significantly more hip flexion, whilst barefoot running was linked with significantly more flexion at the knee and plantarflexion at the ankle. The examination of muscle kinetics indicated that peak forces from Rectus femoris, Vastus medialis, Vastus lateralis, Tibialis anterior were significantly larger in the shod condition whereas Gastrocnemius forces were significantly larger during barefoot running. These observations provide further insight into the mechanical alterations that runners make when running without shoes. Such findings may also deliver important information to runners regarding their susceptibility to chronic injuries in different footwear conditions.

## Introduction

In recent years, interest in barefoot running has expanded and the concept has received considerable research attention (Squadrone and Gallozzo, 2009; Liebermann et al., 2010; Sinclair et al., 2013a b). The interest in barefoot running is based, firstly, around the supposition from evolutionary scientists that running without shoes is the most natural form, as traditional running shoes were not adopted until the 1970’s (Lieberman et al., 2010). Furthermore, clinical interest into barefoot running is based on the consensus that conventional running shoes are associated with an increased frequency of chronic injuries (Lieberman et al., 2010; Robbins and Hanna, 1987).

The mechanics of running barefoot and with shoes have been examined extensively in recent years. Despite this expansion in the literature base, a consensus has yet to be reached regarding the clinical efficacy of running without shoes. The majority of studies in this area have examined kinetic and kinematic biomechanical parameters linked to the aetiology of injury in runners. Research by Liebermann et al. (2010), Hamill et al. (2011) and Squadrone and Gallozi (2009) demonstrated that impact collision forces were significantly reduced as a function of barefoot running. Conversely Sinclair et al. (2013a b) showed that impact loading magnitude was significantly larger during barefoot running compared to running in conventional running shoes. In addition Sinclair (2014) showed that barefoot running was associated with significant reductions in patellofemoral loads, and also linked with a corresponding increase in forces experienced by the Achilles tendon in comparison to conventional footwear.

Whilst the mechanical differences between the two running modalities have received considerable interest, less attention has been paid to the variations in muscle function when running with and without shoes. Olin and Gutierrez (2013) investigated tibialis anterior and medial Gastrocnemius activation magnitude during barefoot and shod running. Activation of the Gastrocnemius was shown to be significantly larger when running without shoes. Shih et al. (2013) examined differences in activation of Rectus femoris, Tibialis anterior, Biceps femoris, and Gastrocnemius muscles when running barefoot and in conventional running trainers. It was demonstrated that activation of the Gastrocnemius was significantly larger when using a forefoot strike pattern. In addition it was also shown that activation of the Tibialis anterior was significantly larger when a rearfoot strike was utilized. Whilst the mechanics of barefoot and shod running have been examined extensively, there has yet to be a comparative examination of the muscles forces associated with the two running modalities, notably with regard to skeletal muscle force distributions.

Recently, bespoke software has been developed which allows skeletal muscle force distributions to be predicted during movement, using motion capture and force platform based data (Delp et al., 2007). To date, such estimations have not been reported during barefoot and shod running. The aim of the current investigation was to examine the differences in the forces produced by the different skeletal muscles during barefoot and shod running. A study of this nature may provide important information regarding the extent of recruitment of the key muscles when running with and without shoes.

## Material and Methods

### Participants

The procedure utilized for this investigation was approved by the Ethics Committee at the University of Central Lancashire. Twenty male participants took part in the current research. The subjects were experienced runners who trained at least 3 times per week. All were free from lower extremity injuries at the time of data collection and provided written informed consent in accordance with the principles outlined in the declaration of Helsinki. The mean anthropometric characteristics of the participants were: age = 27.71 ± 3.01 years, body height = 1.79 ± 0.05 m, body mass = 72.02 ± 4.10 kg.

### Procedure

Participants ran at 4.0 m·s^−1^ (±5%), striking an embedded piezoelectric force platform (Kistler, Kistler Instruments Ltd., Alton, Hampshire) with their right foot (Sinclair et al., 2014). Running velocity was monitored using infrared timing gates (Newtest, Oy Koulukatu, Finland). The stance phase was delineated as the duration over which 20 N or greater of vertical force was applied to the force platform (Sinclair et al., 2013c). Runners completed a minimum of five successful trials in each footwear condition. Kinematics and ground reaction forces data were synchronously collected. Kinematic data were captured at 250 Hz via an eight camera motion analysis system (Qualisys Medical AB, Goteburg, Sweden). Dynamic calibration of the motion capture system was performed before each data collection session.

To define the anatomical frames of the thorax, pelvis, thighs, shanks and feet, retroreflective markers were placed at the C7, T12 and xiphoid process landmarks were also positioned bilaterally onto the acromion process, iliac crest, anterior superior iliac spine, posterior super iliac spine, medial and lateral malleoli, medial and lateral femoral epicondyles and greater trochanter. Carbon-fibre tracking clusters that comprised of four non-linear retroreflective markers were positioned onto the thigh and shank segments. Static calibration trials were obtained with the participant in the anatomical position in order for the positions of the anatomical markers to be referenced in relation to the tracking clusters/markers. A static trial was conducted with the participant in the anatomical position in order for the anatomical positions to be referenced in relation to the tracking markers, following which those not required for dynamic data were removed.

### Data processing

Dynamic trials were digitized using Qualisys Track Manager in order to identify anatomical and tracking markers, then exported as C3D files to Visual 3D (C-Motion, Germantown, MD, USA). Ground reaction force and kinematic data were smoothed using cut-off frequencies of 25 and 12 Hz with a low-pass Butterworth 4th order zero lag filter.

**Figure 1:**
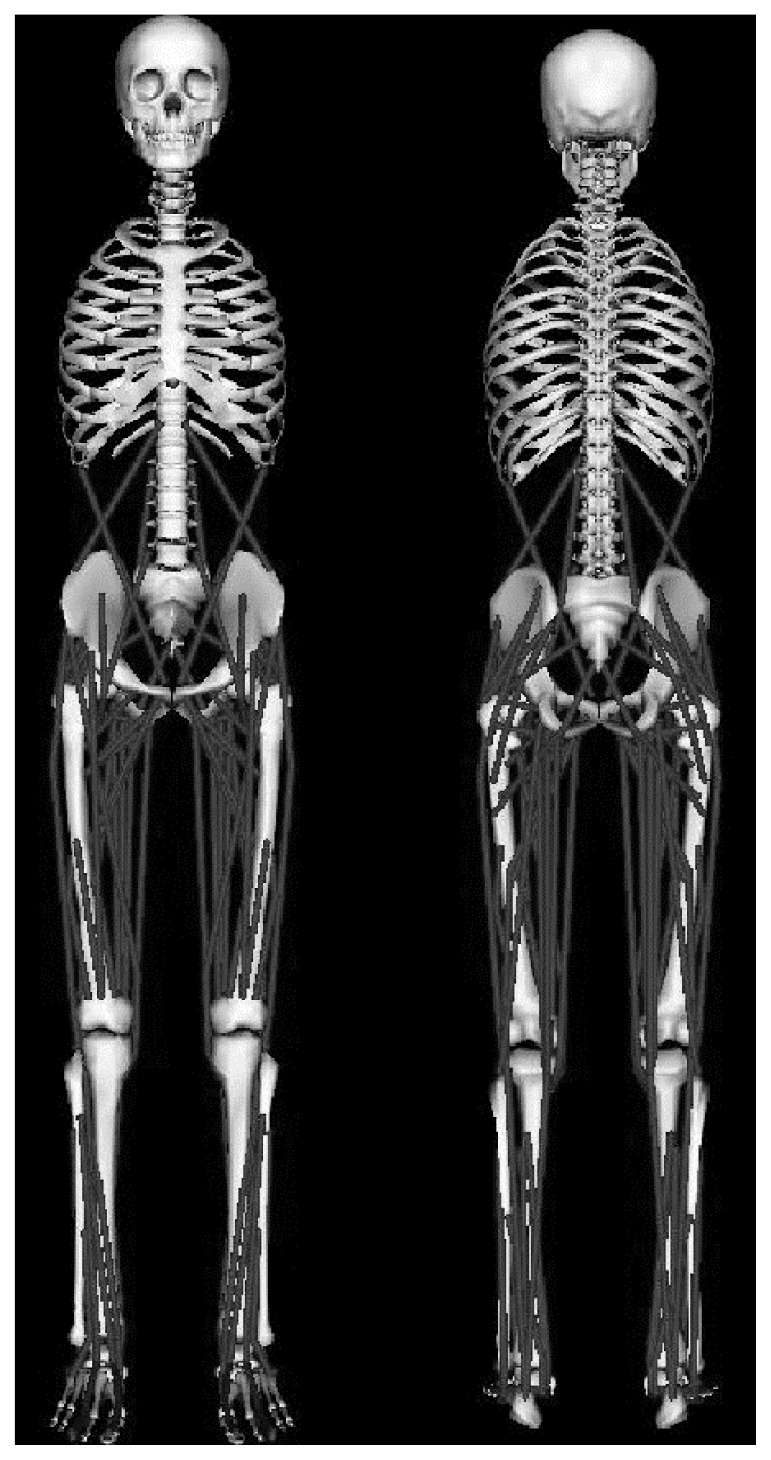
The anterior and posterior view of the OpenSim gait2392 model.

The data during the stance phase were exported from Visual 3D to OpenSim software (Simtk.org, Stanford USA), which was utilized to quantify muscle forces during barefoot and shod running. Simulations of muscle forces were undertaken using the generic gait2392 model within Opensim v3.2 (Figure 1). This model corresponds to the eight segments that were exported from Visual 3D and features 19 total degrees of freedom and 92 muscle-tendon actuators. The muscle intrinsic properties were modelled using the Hill recommendations based on the links between force-velocity-length (Zajac, 1989). These muscle properties were scaled for each individual based on the recommendations of Delp et al. (1990). Following this a residual reduction algorithm (RRA) was employed within OpenSim, this utilized the inverse kinematics and ground reaction forces that were exported from Visual 3D. The RRA calculates the joint torques required to re-create the dynamic motion. The RRA calculations produced route mean squared errors <2°, which correspond with the recommendations for good quality data. Following the RRA, the computed muscle control (CMC) procedure was then employed to estimate a set of muscle force patterns allowing the model to replicate the required kinematics (Thelen et al., 2003). The CMC procedure works by estimating the required muscle forces to produce the net joint torques.

Following the CMC procedure, peak forces during the stance phase were estimated for the Semimembranosus, Semitendinosus, Biceps femoris long head, Biceps femoris short head, Rectus femoris, Vastus medialis, Vastus intermedius, Vastus lateralis, medial Gastrocnemius, lateral Gastrocnemius, Soleus, Tibialis posterior, Tibialis anterior muscles from the right side. The net peak muscle force values were normalized by dividing by the participants’ body mass, thus allowing muscles forces to be expressed as N·kg. Sagittal plane kinematic parameters were calculated using an XYZ cardan sequence of rotations (where X represents sagittal plane, Y represents coronal plane and Z represents transverse plane rotations). Kinematic parameters measures which were extracted for statistical analysis were 1) the angle at footstrike, 2) the peak angle during stance and 3) the relative range of motion (ROM) from footstrike to the peak angle.

### Footwear

The shod condition during this study consisted of a Saucony Pro Grid Guide II running trainers. The shoes were the same for all runners; they differed in size only (sizes 8–10 in men’s shoe UK sizes).

### Statistical analyses

Descriptive statistics (means and standard deviations) were obtained for each footwear condition. Shapiro-Wilk tests were used to screen the data for normality. Depending on whether the data exhibited a normal distribution, footwear mediated differences in kinetics and sagittal plane kinematic parameters were examined using either repeated measures or Friedman’s ANOVA. To control type I error, statistical significance was accepted at the p<0.05 level (Sinclair et al., 2013d). Effect sizes for all significant findings were calculated using partial Eta^2^ (pη^2^). All statistical actions were conducted using SPSS v22.0 (SPSS Inc, Chicago, USA).

## Results

**Figure 2:**
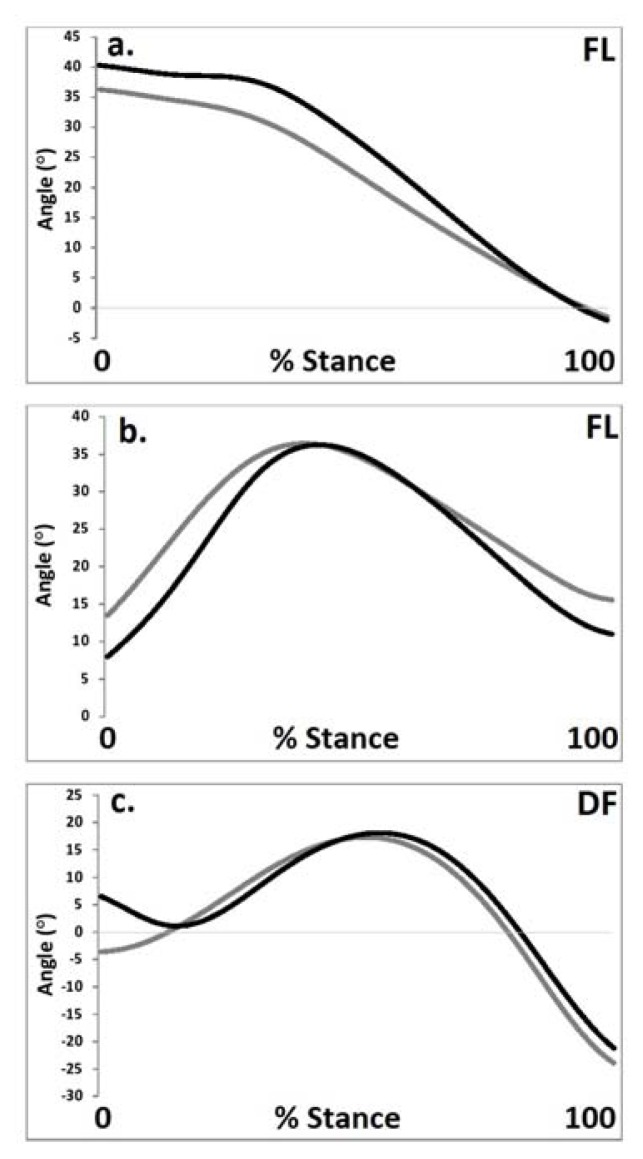
Sagittal plane kinematics of the a. hip, b. knee and c. ankle as a function of the different footwear conditions (black = shod, grey = barefoot) (FL = flexion, DF = dorsiflexion).

Table 1 and Figure 2 present the muscle force distributions and hip, knee and thorax kinematics obtained as a function of different squat techniques. The results indicate that whilst the kinematic curves from the two conditions were qualitatively similar, squat technique significantly affected the outcome muscle kinetics and joint kinematics.

### Joint kinematics

For the hip joint the degree of flexion at footstrike was significantly (F _(19)_ = 9.56, p<0.05, pη^2^ = 0.48) larger during the shod condition. Furthermore peak hip flexion was also shown to be significantly (F _(19)_ = 9.02, p<0.05, pη^2^ = 0.45) larger during the shod condition. For the knee joint the degree of flexion at footstrike was significantly (X^2^_(1)_ = 12.88, p<0.05, pη^2^ = 0.50) larger when running barefoot. In addition, a relative ROM was also shown to be significantly (F _(19)_ = 15.21, p<0.05, pη^2^ = 0.64) larger during the shod condition. For the ankle joint in the barefoot condition the degree of plantarflexion at footstrike was significantly (F _(19)_ = 20.33, p<0.05, pη^2^ = 0.70) larger than during the shod condition. In addition, a relative ROM was shown to be significantly (F _(19)_ = 13.74, p<0.05, pη^2^ = 0.59) larger during the barefoot condition.

### Predicted muscle kinetics

The results show that peak forces in the Rectus femoris (F _(19)_ = 9.22, p<0.05, pη^2^ = 0.46), Vastus medialis (F _(19)_ = 9.88, p<0.05, pη^2^ = 0.48) and Vastus lateralis (F _(19)_ = 17.51, p<0.05, pη^2^ = 0.66) muscles were significantly larger in the shod condition. In addition to this, peak medial Gastrocnemius force was shown to be significantly (F _(19)_ = 13.33, p<0.05, pη^2^ = 0.56) larger in the barefoot condition. Finally it was demonstrated that peak Tibialis anterior forces were significantly (F _(19)_ = 17.65, p<0.05, pη^2^ = 0.67) greater in the shod condition compared to running barefoot.

## Discussion

The current study investigated the influence of the barefoot and shod running techniques on the sagittal plane kinematics and forces produced by skeletal muscles. This represents the first comparative study to simultaneously examine differences in sagittal plane kinematics and muscles force production when running with and without shoes.

The first key observation from the current investigation is that the hip joint was shown to exhibit significantly more flexion in the shod condition in comparison to barefoot. It is likely that this observation relates to the reduced stride lengths that have been found to be associated with barefoot running (Sinclair et al., 2013a b). In addition to this it was also demonstrated that knee flexion at footstrike was significantly larger in the barefoot condition and the knee relative ROM was significantly increased during shod running. These kinematic observations may serve to explain the mechanism by which increased quadriceps forces were demonstrated during shod running compared to barefoot. A reduced stride length has the effect of positioning the stance limb closer to the centre of mass, which has been shown to reduce the amount of work output generated by the quadriceps during the impact phase of running (Arendse et al., 2004). Therefore because the requirements of the knee joint as an energy absorber are lessened, the extent of the knee relative ROM is also reduced. The quadriceps moment arm is inversely related to the knee flexion range (van Eijden, 1986), thus, a larger quadriceps force output was required in the shod condition to overcome this mechanical disadvantage exhibited when running without shoes (Sinclair, 2014).

This finding may also be able to provide important clinical information regarding the aetiology of injury in runners. Increased quadriceps forces have been shown, through musculoskeletal modelling, to be positively associated with the loads experienced by the patellofemoral joint during dynamic tasks (LaBella, 2004). Patellofemoral pain is the most common chronic pathology in recreational runners (van Gent et al., 2007). The widely accepted consensus regarding aetiology of patellofemoral pathology is that the symptoms originate as a function of excessive patellofemoral joint kinetics (Kulmala et al., 2013). Taking into account the incidence of patellofemoral pathology in runners, this study provides additional support to the propositions of Sinclair (2014) and Kulmala et al. (2013) that running without shoes may be a mechanism by which the incidence of patellofemoral pathologies may be attenuated.

The second key observation is in relation to the ankle joint, which was shown to be significantly more plantar flexed when running barefoot in comparison to the shod condition. This finding agrees with those of Lieberman et al. (2010) and Sinclair et al. (2013a b), who also noted increases in plantar flexion when running without shoes and indicates that runners adopted a mid/forefoot running pattern in the barefoot condition. These findings may serve to explain the mechanisms by which increases in peak Gastrocnemius and Tibialis anterior forces were observed in the barefoot and shod conditions, respectively. In the barefoot condition the ankle was shown to exhibit plantarflexion at footstrike whilst during shod running runners landed with the ankle in a dorsiflexed position. The Gastrocnemius and Tibialis anterior are primary contributors to both plantar and dorsiflexion, thus, it is to be expected that these muscles would exhibit increases in force output in order to facilitate sagittal plane movement of the ankle joint in the appropriate direction. These observations support those of Sinclair (2014) and Kulmala et al. (2013) who noted the presence of a higher ankle contribution to the deceleration of the body when running without shoes.

It is important that the observations from the current investigation be contextualised taking into account the increased stride frequencies typically observed during barefoot running. Increased stride frequency is associated with reductions in energy absorption during the impact phase of running (Sinclair, 2014). Therefore whilst increases in quadriceps muscle forces were noted per step during shod running, the amount of cumulative force output and potential patellofemoral stress may not be affected between different footwear conditions, as the total number of footfalls required to achieve the same velocity/distance is also correspondingly increased. There is currently a lack of published information regarding the effects of cumulative and singular loads on musculoskeletal structures during running. Thus it is recommended that future studies prospectively examine the effects of running with and without shoes in terms of their propensity for chronic injury development.

As the current investigation used a simulation based procedure to estimate muscle kinetics this may serve as a limitation. The effectiveness of musculoskeletal simulations is reliant on the calculations undertaken by the mathematical model itself. Numerous assumptions have been made in the construction of musculoskeletal simulation models (Delp et al., 1990). These relate to rotational constraints placed on the knee and ankle joints and absence of muscles such as the recuts abdominis, which may lead to incorrectly predicted muscle forces. However, as direct measurements of muscle kinetics are currently not possible the current procedure represents the most practicable technique for the investigation of muscle forces in dynamic movements. It may therefore be prudent for future analyses to consider differences in activation of the key muscles associated with running as a function of barefoot and shod locomotion.

In conclusion, although previous analyses have comparatively examined the mechanics of barefoot and shod running, the current knowledge with regard to the differences in muscles forces between the two modalities is limited. The present investigation addresses this topic by providing a comparison of lower extremity muscles forces and sagittal plane kinematics when running with and without shoes. The current study shows that the shod condition was associated with significantly more hip flexion, whilst barefoot running was linked with significantly more flexion at the knee and plantarflexion at the ankle. The muscle kinetics indicated that peak forces from Rectus femoris, Vastus medialis, Vastus lateralis and Tibialis anterior were significantly larger in the shod condition, whereas Gastrocnemius forces were significantly larger during barefoot running. These observations provide further insight into the mechanical alterations that runners make when running without shoes and may also deliver important clinical information to runners regarding their susceptibility to chronic injuries in different footwear conditions.

## Figures and Tables

**Table 1:** Sagittal plane kinematics as a function of barefoot and shod running conditions

	Shod		Barefoot	

	Mean	*SD*	Mean	*SD*
Hip				
Angle at footstrike (°)	40.84	*4.09*	36.08	*3.74*
Peak flexion (°)	41.92	*4.25*	37.21	*4.11*
Relative range of motion (°)	1.08	*1.00*	1.13	*0.98*
Knee				
Angle at footstrike (°)	7.56	*5.03*	13.83	*4.87*
Peak flexion (°)	35.27	*5.74*	35.66	*5.26*
Relative range of motion (°)	27.71	*2.08*	21.83	*1.82*
Ankle				
Angle at footstrike (°)	7.12	*6.01*	−3.97	*6.58*
Peak dorsiflexion (°)	16.28	*4.28*	15.59	*4.69*
Relative range of motion (°)	9.16	*3.27*	19.56	*3.24*

**Table 2:** Peak muscle forces as a function of barefoot and shod running conditions

	Shod		Barefoot	

	Mean	SD	Mean	SD
Semimembranosus (N·kg)	5.43	*2.89*	3.85	*1.53*
Semitendinosus (N·kg)	1.90	*1.09*	1.47	*0.86*
Biceps femoris long head (N·kg)	4.69	*1.94*	3.49	*1.39*
Biceps femoris short head (N·kg)	3.37	*1.89*	3.12	*2.45*
Rectus femoris (N·kg)	7.48	*5.83*	5.23	*4.59*
Vastus medialis (N·kg)	8.41	*3.09*	6.28	*2.55*
Vastus intermedius (N·kg)	9.63	*3.68*	8.31	*3.93*
Vastus lateralis (N·kg)	16.88	*6.86*	12.98	*5.37*
Medial Gastrocnemius (N·kg)	11.36	*2.09*	14.99	*3.22*
Lateral Gastrocnemius (N·kg)	5.66	*1.28*	7.14	*1.77*
Soleus (N·kg)	43.08	*9.72*	45.30	*12.02*
Tibialis posterior (N·kg)	17.20	*5.04*	18.70	*3.63*
Tibialis anterior (N·kg)	6.07	*2.08*	3.92	*2.76*
